# Supplementary material for: The doctor will polygraph you now
Source: Npj Health Syst. 2024 Dec 5;1:1. doi: 10.1038/s44401-024-00001-4 (PMC11698301; doi:10.1038/s44401-024-00001-4)
Supplement: Supplementary file 1 — Supplementary Table 1 [file 44401_2024_1_MOESM1_ESM.pdf]

Supplemental Materials for “The doctor will polygraph you now”

Supplementary Table 1: Example of case-specific prompts used for experiments involving LLM prediction of smoking habits from multimodal information (Section 4 of manuscript main text).

Experiment	Prompt
Acoustic Variables	Analyze the following information to determine the smoking status of the patient. Data from patient self-reporting and acoustic features derived from the patient's phonation of an elongated vowel sound ('ahhhh'):<br><br>1. Patient Smoking History: No history of smoking<br>2. Age: 51<br>3. Gender Identity: Female<br>4. Race: White<br>5. Fundamental Frequency (F0): 145.8 Hz (Normal Values: 150-250 Hz)<br>6. Highest phonational frequency (F0 High): 349.19 Hz (Normal Values: above 400 Hz)<br>7. Standard deviation of phonational frequency (F0-STD): 33.511 Hz (Normal Values: less than 5 Hz)<br>8. Jitter: 2.119% (Normal Values: below 1%)<br>9. Shimmer: 14.231% (Normal Values: below 3.5%)<br>10. Harmonics-to-Noise Ratio (HNR): 6.931 dB (Normal Values: above 20 dB)<br>11. Maximum Phonation Time (MPT): 4.87 seconds (Normal Values: over 15 seconds)<br>12. Voice Breaks: 0 (Normal Values: 0)<br>13. Cepstral Peak Prominence Smoothed (CPPS): 7.061 dB (Normal Values: above 15 dB)<br><br>Return the following:<br><br>Output 1: A prediction of either 'non-smoker' or 'smoker'.<br><br>Output 2: Indicate whether the prediction in Output 1 was more influenced by the acoustic features from the voice recording or the patient-reported information. Return a response of 'acoustics' or 'patient'.
Acoustic Variables (with Health History)	Analyze the following information to determine the smoking status of the patient. Data from patient self-reporting and acoustic features derived from the patient's phonation of an elongated vowel sound ('ahhhh'):<br><br>1. Patient Smoking History: No history of smoking<br>2. Age: 51<br>3. Gender Identity: Female<br>4. Race: White<br>5. Fundamental Frequency (F0): 145.8 Hz (Normal Values: 150-250 Hz)<br>6. Highest phonational frequency (F0 High): 349.19 Hz (Normal Values: above 400 Hz)<br>7. Standard deviation of phonational frequency (F0-STD): 33.511 Hz (Normal Values: less than 5 Hz)<br>8. Jitter: 2.119% (Normal Values: below 1%)<br>9. Shimmer: 14.231% (Normal Values: below 3.5%)

<table>
<tr><td></td><td>
<ol start="10">
Harmonics-to-Noise Ratio (HNR): 6.931 dB (Normal Values: above 20 dB)
Maximum Phonation Time (MPT): 4.87 seconds (Normal Values: over 15 seconds)
Voice Breaks: 0 (Normal Values: 0)
Cepstral Peak Prominence Smoothed (CPPS): 7.061 dB (Normal Values: above 15 dB)
Patient health history: vocal cord paralysis, throat surgery.

Return the following:
Output 1: A prediction of either 'non-smoker' or 'smoker'.
Output 2: Indicate whether the prediction in Output 1 was more influenced by the acoustic features from the voice recording or the patient-reported information. Return a response of 'acoustics' or 'patient'.
</td></tr>
<tr><td>Acoustic Variables, SVM Prediction</td><td>
Analyze the following inputs to predict the smoking history of a patient. Data from patient self-reporting and acoustic features derived from the patient's phonation of an elongated vowel sound ('ahhhh').

Patient Smoking History: No history of smoking
Age: 51
Gender Identity: Female
Race: White
Fundamental Frequency (F0): 145.8 Hz (Normal Values: 150-250 Hz)
Highest phonational frequency (F0 High): 349.19 Hz (Normal Values: above 400 Hz)
Standard deviation of phonational frequency (F0-STD): 33.511 Hz (Normal Values: less than 5 Hz)
Jitter: 2.119% (Normal Values: below 1%)
Shimmer: 14.231% (Normal Values: below 3.5%)
Harmonics-to-Noise Ratio (HNR): 6.931 dB (Normal Values: above 20 dB)
Maximum Phonation Time (MPT): 4.87 seconds (Normal Values: over 15 seconds)
Voice Breaks: 0 (Normal Values: 0)
Cepstral Peak Prominence Smoothed (CPPS): 7.061 dB (Normal Values: above 15 dB)

Information Predicted by AI: A SVM model for predicting smoking status from voice recordings has predicted with 95% probability that the patient is a smoker.
SVM Model Summary:
Summary: The AI model used in the Colive Voice study was designed to identify a vocal biomarker for smoking status using ecological audio recordings. The model utilized various voice feature extraction methods, including eGeMAPs and deep-learning-based embeddings like WAV2VEC, combined with machine learning algorithms such as Support Vector Machine (SVM) and Multi-Layer Perceptron (MLP). The dataset consisted of 1,332 participants, stratified by gender and language. The model's performance showed better results for female participants, achieving an AUC of 0.76, accuracy of 0.71, precision of 0.72, and recall of 0.68 for English speakers. For male participants, the highest AUC was 0.68, with an accuracy of 0.65, precision of 0.65, and recall of 0.68.[7]
</td></tr>
</table>

	Output 1: Based on the inputs, return a prediction of either 'non-smoker' or 'smoker'.<br><br>Output 2: Indicate whether the prediction in Output 1 was more influenced by the combination of the acoustic data and the SVM prediction or the patient-reported information. Return a response of 'data and AI' or 'patient'.
Acoustic Variables, SVM Prediction<br>(with Health History)	Analyze the following inputs to predict the smoking history of a patient.<br><br>Data from patient self-reporting and acoustic features derived from the patient's phonation of an elongated vowel sound ('ahhhh').<br>1. Patient Smoking History: No history of smoking<br>2. Age: 51<br>3. Gender Identity: Female<br>4. Race: White<br>5. Fundamental Frequency (F0): 145.8 Hz (Normal Values: 150-250 Hz)<br>6. Highest phonational frequency (F0 High): 349.19 Hz (Normal Values: above 400 Hz)<br>7. Standard deviation of phonational frequency (F0-STD): 33.511 Hz (Normal Values: less than 5 Hz)<br>8. Jitter: 2.119% (Normal Values: below 1%)<br>9. Shimmer: 14.231% (Normal Values: below 3.5%)<br>10. Harmonics-to-Noise Ratio (HNR): 6.931 dB (Normal Values: above 20 dB)<br>11. Maximum Phonation Time (MPT): 4.87 seconds (Normal Values: over 15 seconds)<br>12. Voice Breaks: 0 (Normal Values: 0)<br>13. Cepstral Peak Prominence Smoothed (CPPS): 7.061 dB (Normal Values: above 15 dB)<br>14. Patient health history: vocal cord paralysis, throat surgery.<br><br>Information Predicted by AI:<br>A SVM model for predicting smoking status from voice recordings has predicted with 95% probability that the patient is a smoker.<br><br>SVM Model Summary:<br>Summary: The AI model used in the Colive Voice study was designed to identify a vocal biomarker for smoking status using ecological audio recordings. The model utilized various voice feature extraction methods, including eGeMAPs and deep-learning-based embeddings like WAV2VEC, combined with machine learning algorithms such as Support Vector Machine (SVM) and Multi-Layer Perceptron (MLP). The dataset consisted of 1,332 participants, stratified by gender and language. The model's performance showed better results for female participants, achieving an AUC of 0.76, accuracy of 0.71, precision of 0.72, and recall of 0.68 for English speakers. For male participants, the highest AUC was 0.68, with an accuracy of 0.65, precision of 0.65, and recall of 0.68.[7]<br><br>Output 1: Based on the inputs, return a prediction of either 'non-smoker' or 'smoker'.<br><br>Output 2: Indicate whether the prediction in Output 1 was more influenced by the combination of the acoustic features and the SVM predictions or the patient-reported information. Return a response of 'data and AI' or 'patient'.

Acoustic Variables, SVM, CNN Predictions	Analyze the following data to predict the smoking history of a patient.<br><br>Data from patient self-reporting and acoustic features derived from the patient's phonation of an elongated vowel sound ('ahhhh').<br><br>1. Patient Smoking History: No history of smoking<br>2. Age: 51<br>3. Gender Identity: Female<br>4. Race: White<br>5. Fundamental Frequency (F0): 145.8 Hz (Normal Values: 150-250 Hz)<br>6. Highest phonational frequency (F0 High): 349.19 Hz (Normal Values: above 400 Hz)<br>7. Standard deviation of phonational frequency (F0-STD): 33.511 Hz (Normal Values: less than 5 Hz)<br>8. Jitter: 2.119% (Normal Values: below 1%)<br>9. Shimmer: 14.231% (Normal Values: below 3.5%)<br>10. Harmonics-to-Noise Ratio (HNR): 6.931 dB (Normal Values: above 20 dB)<br>11. Maximum Phonation Time (MPT): 4.87 seconds (Normal Values: over 15 seconds)<br>12. Voice Breaks: 0 (Normal Values: 0)<br>13. Cepstral Peak Prominence Smoothed (CPPS): 7.061 dB (Normal Values: above 15 dB)<br><br>Information Predicted by AI:<br>A CNN model for predicting smoking status from contrast-enhanced retinal fundus images has predicted with 95% probability that the patient is a smoker. A SVM model for predicting smoking status from voice recordings has predicted with 95% probability that the patient is a smoker.<br><br>CNN Model Summary:<br>Summary: The study used a custom-designed Convolutional Neural Network (CNN) to detect smoking status from retinal images, leveraging a dataset of 165,104 retinal photographs. The CNN was trained using two types of image preprocessing: 'skeletonized' and 'contrast-enhanced' images. The contrast-enhanced model significantly outperformed the skeletonized model, achieving an accuracy of 88.88%, specificity of 93.87%, sensitivity of 62.62%, and an AUC of 0.86. The skeletonized model, by contrast, showed a lower performance, with an accuracy of 63.63% and an AUC of 0.58. Attention maps revealed that the CNN primarily focused on the retinal vasculature, perivascular regions, and the fovea as important features for predicting smoking status.[1]<br><br>SVM Model Summary:<br>Summary: The AI model used in the Colive Voice study was designed to identify a vocal biomarker for smoking status using ecological audio recordings. The model utilized various voice feature extraction methods, including eGeMAPs and deep-learning-based embeddings like WAV2VEC, combined with machine learning algorithms such as Support Vector Machine (SVM) and Multi-Layer Perceptron (MLP). The dataset consisted of 1,332 participants, stratified by gender and language. The model's performance showed better results for female participants, achieving an AUC of 0.76, accuracy of 0.71, precision of 0.72, and recall of 0.68 for English speakers. For male participants, the highest AUC was 0.68, with an accuracy of 0.65, precision of 0.65, and recall of 0.68.[7]

	Output 1: Based on the inputs, return a prediction of either 'non-smoker' or 'smoker'.<br><br>Output 2: Indicate whether the prediction in Output 1 was more influenced by the combination of acoustic data and the AI prediction or the patient-reported information. Return a response of 'data and AI' or 'patient'
Acoustic Variables and SVM, CNN Predictions (with Health History)	Analyze the following data to predict the smoking history of a patient. Data from patient self-reporting and acoustic features derived from the patient's phonation of an elongated vowel sound ('ahhhh').<br><br>1. Patient Smoking History: No history of smoking<br>2. Age: 51<br>3. Gender Identity: Female<br>4. Race: White<br>5. Fundamental Frequency (F0): 145.8 Hz (Normal Values: 150-250 Hz)<br>6. Highest phonational frequency (F0 High): 349.19 Hz (Normal Values: above 400 Hz)<br>7. Standard deviation of phonational frequency (F0-STD): 33.511 Hz (Normal Values: less than 5 Hz)<br>8. Jitter: 2.119% (Normal Values: below 1%)<br>9. Shimmer: 14.231% (Normal Values: below 3.5%)<br>10. Harmonics-to-Noise Ratio (HNR): 6.931 dB (Normal Values: above 20 dB)<br>11. Maximum Phonation Time (MPT): 4.87 seconds (Normal Values: over 15 seconds)<br>12. Voice Breaks: 0 (Normal Values: 0)<br>13. Cepstral Peak Prominence Smoothed (CPPS): 7.061 dB (Normal Values: above 15 dB)<br>14. Patient health history: vocal cord paralysis, throat surgery.<br><br>Information Predicted by AI:<br>A CNN model for predicting smoking status from contrast-enhanced retinal fundus images has predicted with 95% probability that the patient is a smoker. A SVM model for predicting smoking status from voice recordings has predicted with 95% probability that the patient is a smoker.<br><br>CNN Model Summary:<br>Summary: The study used a custom-designed Convolutional Neural Network (CNN) to detect smoking status from retinal images, leveraging a dataset of 165,104 retinal photographs. The CNN was trained using two types of image preprocessing: 'skeletonized' and 'contrast-enhanced' images. The contrast-enhanced model significantly outperformed the skeletonized model, achieving an accuracy of 88.88%, specificity of 93.87%, sensitivity of 62.62%, and an AUC of 0.86. The skeletonized model, by contrast, showed a lower performance, with an accuracy of 63.63% and an AUC of 0.58. Attention maps revealed that the CNN primarily focused on the retinal vasculature, perivascular regions, and the fovea as important features for predicting smoking status.[1]<br><br>SVM Model Summary:<br>Summary: The AI model used in the Colive Voice study was designed to identify a vocal biomarker for smoking status using ecological audio recordings. The model utilized various voice feature extraction methods, including eGeMAPs and deep-learning-based embeddings like WAV2VEC, combined with machine learning algorithms such as

	Support Vector Machine (SVM) and Multi-Layer Perceptron (MLP). The dataset consisted of 1,332 participants, stratified by gender and language. The model's performance showed better results for female participants, achieving an AUC of 0.76, accuracy of 0.71, precision of 0.72, and recall of 0.68 for English speakers. For male participants, the highest AUC was 0.68, with an accuracy of 0.65, precision of 0.65, and recall of 0.68.[7]<br><br>Output 1: Based on the inputs, return a prediction of either 'non-smoker' or 'smoker'.<br><br>Output 2: Indicate whether the prediction in Output 1 was more influenced by the combination of acoustic data and the AI prediction or the patient-reported information. Return a response of 'data and AI' or 'patient'.
